# Detection of cerebrospinal fluid biomarkers changes of Alzheimer’s disease using a cognitive stress test in persons with subjective cognitive decline and mild cognitive impairment

**DOI:** 10.3389/fpsyg.2024.1373541

**Published:** 2024-06-26

**Authors:** Maria Valles-Salgado, María José Gil-Moreno, Rosie E. Curiel Cid, Alfonso Delgado-Álvarez, Isabel Ortega-Madueño, Cristina Delgado-Alonso, Marta Palacios-Sarmiento, Juan I. López-Carbonero, María Cruz Cárdenas, Jorge Matías-Guiu, María Díez-Cirarda, David A. Loewenstein, Jordi A. Matias-Guiu

**Affiliations:** ^1^Department of Neurology, Hospital Clínico San Carlos, San Carlos Health Research Institute (IdISSC), Universidad Complutense de Madrid, Madrid, Spain; ^2^Center for Cognitive Neuroscience and Aging, Department of Psychiatry and Behavioral Sciences, Miller School of Medicine, University of Miami Miller School of Medicine, Miami, FL, United States; ^3^Department of Clinical Analysis, Institute of Laboratory, Hospital Clínico San Carlos, San Carlos Health Research Institute (IdISSC), Universidad Complutense de Madrid, Madrid, Spain

**Keywords:** Alzheimer’s disease, semantic interference, memory, mild cognitive impairment, neuropsychological assessment, cerebrospinal fluid biomarkers

## Abstract

**Introduction:**

Timely and accurate diagnosis of the earliest manifestations of Alzheimer’s disease (AD) is critically important. Cognitive challenge tests such as the Loewenstein Acevedo Scales for Semantic Interference and Learning (LASSI-L) have shown favorable diagnostic properties in a number of previous investigations using amyloid or FDG PET. However, no studies have examined LASSI-L performance against cerebrospinal fluid biomarkers of AD, which can be affected before the distribution of fibrillar amyloid and other changes that can be observed in brain neuroimaging. Therefore, we aimed to evaluate the relationship between LASSI-L scores and CSF biomarkers and the capacity of the cognitive challenge test to detect the presence of amyloid and tau deposition in patients with subjective cognitive decline and amnestic mild cognitive impairment (MCI).

**Methods:**

One hundred and seventy-nine patients consulting for memory loss without functional impairment were enrolled. Patients were examined using comprehensive neuropsychological assessment, the LASSI-L, and cerebrospinal fluid (CSF) biomarkers (Aβ1-42/Aβ1-40 and ptau181). Means comparisons, correlations, effect sizes, and ROC curves were calculated.

**Results:**

LASSI-L scores were significantly associated with CSF biomarkers Aβ1-42/Aβ1-40 in patients diagnosed with MCI and subjective cognitive decline, especially those scores evaluating the capacity to recover from proactive semantic interference effects and delayed recall. A logistic regression model for the entire sample including LASSI-L and age showed an accuracy of 0.749 and an area under the curve of 0.785 to detect abnormal amyloid deposition.

**Conclusion:**

Our study supports the biological validity of the LASSI-L and its semantic interference paradigm in the context of the early stages of AD. These findings emphasize the utility and the convenience of including sensitive cognitive challenge tests in the assessment of patients with suspicion of early stages of AD.

## 1 Introduction

Alzheimer’s disease (AD) is a neurodegenerative disorder pathologically characterized by the presence of beta-amyloid plaques and tau neurofibrillary tangles (Braak and Braak, 1991). The first pathophysiological events of the disease often begin many years before the development of dementia, and patients slowly progress from absent or minimal symptoms (preclinical stages) to mild cognitive impairment (prodromal AD), when the first cognitive deficits are typically identified. Eventually, the dementia syndrome or Major Neurocognitive Disorder by DSM-5 criteria occurs (American Psychiatric Association, 2022), which is characterized by clear functional impairment. Recently, the first disease pathology modifying therapies have shown some efficacy (van Dyck et al., 2023). However, one of the most challenging issues in the field remains the early and accurate diagnosis of AD, since the success of the earliest treatments are dependent on capturing the disease before the occurrence of multi-system degeneration or the “Alzheimer’s cascade” (Jucker and Walker, 2023).

Better clinical characterization of patients at the earliest possible stages of disease represents an important advancement that is needed in the field. For example, patients clinically diagnosed with amnestic MCI (aMCI) are at greater risk than other non-memory phenotypes and this clinical phenotype may signal the prodromal phase of AD. However, even in patients with aMCI, the underlying causes are heterogeneous (Petersen, 2016), and a significant percentage have no amyloid deposition. The capacity of traditional neuropsychological measures to accurately detect the presence of disease states with specificity or predict the progression of illness during the earliest stages has yielded mixed results (Duke Han et al., 2017; Thomas et al., 2018; Pan et al., 2020; Stricker et al., 2020; Kehl-Floberg et al., 2023; Curiel Cid et al., 2023b), particularly when only subtle cognitive decline may be present. Thus, further refinement of clinical and cognitive characterization is necessary for a better stratification of patients with regards to both early cognitive impairment and their suspected etiologies (Bondi et al., 2017; Curiel Cid et al., 2023a).

One of the most relevant advances in the field of neuropsychological assessment has been the development of new and sensitive cognitive tools focused on an early and accurate diagnosis. These tools have been categorized under the term “cognitive stress” or “cognitive challenge tests” (Loewenstein et al., 2018; Curiel Cid et al., 2023b). A more refined characterization of memory deficits could be useful to distinguish patients with amyloid deposition, at least in aMCI (Tomadesso et al., 2019). Combing such measures would provide the ability to track cognitive changes and facilitate early interventions. In this regard, the Loewenstein-Acevedo Scales for Semantic Interference and Learning (LASSI-L) is a novel cognitive stress paradigm that uses controlled learning and cued recall to maximize the storage of a list of 15 words that belong to one of three semantic categories (Crocco et al., 2014). A relevant characteristic of this paradigm is the presentation of a second list of 15 different and semantically competing words. That is, all 15 words on the second list belong to the identical semantic categories as the first list. Both lists are presented twice, allowing to evaluate whether proactive semantic interference impacted performance and, uniquely, whether the patient is able to recover from the effects of proactive semantic interference. This test has shown favorable diagnostic properties for the diagnosis of aMCI and AD dementia (Crocco et al., 2014; Matias-Guiu et al., 2017) and has been associated with grey matter volumes and brain metabolism of regions closely linked to the first stages of AD (Loewenstein et al., 2017; Valles-Salgado et al., 2022). Furthermore, the test scores have been associated with amyloid load in community-dwelling elders (Loewenstein et al., 2016) and participants from an Alzheimer’s Disease Research Center with many cases referred by a specialty memory disorders clinic (Zheng et al., 2022).

In this investigation, we hypothesized that LASSI-L would detect the first pathophysiological events related to amyloid and/or tau deposition in a large cohort of patients with memory complaints with no evidence of functional impairments. Thus, we aimed to evaluate the relationship between the LASSI-L scores and CSF biomarkers and the capacity of the LASSI-L cognitive challenge test to detect the alterations in amyloid and tau CSF biomarkers.

## 2 Methods

### 2.1 Participants

We enrolled patients consulting to our center for memory loss but with no significant functional impairment in daily living activities. All patients were Spaniards, and Spanish was their native language. They were enrolled between January 2019 and June 2023. All patients were examined with a comprehensive neuropsychological protocol and the LASSI-L. The LASSI-L was not used for diagnostic purposes to avoid circularity. For this study, we selected patients with cerebrospinal fluid biomarkers.

Inclusion criteria were as follows: (a) Patients consulting due to memory loss; (b) Absence of functional impairment in daily-living activities (Functional Activities Questionnaire <2) (Olazarán et al., 2005); (c) At least one clinical criterion suggestive of risk of AD (family history of AD; age of onset of cognitive symptoms >60 years-old; clinical characteristics of the memory loss; neuropsychological profile suggestive of amnestic MCI); (d) absence of alternative explanations to memory complaints such as depression, medical or neurological comorbidities with potential cognitive consequences. Exclusion criteria included: (a) Contraindications for lumbar puncture; (b) neurological disorders potentially associated with cognitive impairment or biasing cognitive examination (e.g., stroke, epilepsy, etc.); (c) active psychiatric disease (e.g., depression, bipolar disorder, etc.); (d) substance abuse; (e) visual, auditory, or other sensory impairment that could impair test performance. The main clinical and demographic characteristics are shown in Table 1. The table shows the comparison between the initial and the final sample after excluding those patients with suspected non-Alzheimer’s disease pathophysiology according to the CSF results (abnormal t-tau with amyloid within normal limits) (Jack et al., 2016a).

**Table 1:** Main clinical and demographic characteristics.

	Initial sample	Final sample
Sample size	199	179
Age	70.67 ± 7.06	70.52 ± 7.16
Years of education	11.00 ± 4.75	11.16 ± 4.82
Sex (% Females)	106 (53.3%)	93 (51.95%)
ACE-III	78.47 ± 11.96	78.52 ± 12.32
Aβ1-42/Aβ1-40 ratio	0.074 ± 0.046	0.071 ± 0.031
Aβ1-42 (pg/mL)	958.49 ± 461.88	895.73 ± 409.18
p-tau181 (pg/mL)	77.73 ± 58.60	78.36 ± 61.46
t-tau (pg/mL)	497.65 ± 319.64	495.26 ± 335.81
Aβ1-42/ptau181 ratio	20.12 ± 14.84	19.82 ± 15.26

The final sample was obtained after excluding those patients with suspected non-Alzheimer’s disease pathophysiology according to CSF results. ACE-III, Addenbrooke’s Cognitive Examination.

The local Ethics Committee approved the research protocol, and all participants gave written informed consent to be included in the study.

### 2.2 Neuropsychological assessment

A comprehensive neuropsychological assessment was conducted, which encompassed the Addenbrooke’s Cognitive Examination III (ACE-III) (Matias-Guiu et al., 2015a) and a standardized neuropsychological battery that examines the main cognitive domains and has been co-normed in our setting (Peña-Casanova et al., 2009). The complete protocol has been specified elsewhere (Valles-Salgado et al., 2022). The Free and Cued Selective Reminding Test (FCSRT) was used to categorize patients with aMCI. Briefly, those patients showing a −1.5 SD in the age and education-adjusted scores in at least one of the scores measuring learning or delayed recall (total free recall, total recall, delayed free recall, and delayed total recall) were classified as aMCI. We used the FCSRT for supporting the aMCI diagnosis, because it has been specifically recommended for the diagnosis of prodromal stages of AD (Dubois et al., 2014; Lemos et al., 2015). Those patients that had complained of memory loss but not meeting these criteria (FCSRT within normal limits) were considered as subjective cognitive decline (SCD). The FCSRT was unavailable in four patients.

According to the specified criteria, 103 patients were categorized as MCI and 72 patients as SCD. Patients with MCI showed lower levels than SCD in Aβ1-42/Aβ1-40 (0.064 ± 0.029 vs. 0.077 ± 0.031; *U* = 2,762, *p* = 0.007) and Aβ1-42/ptau181 (16.84 ± 14.15 vs. 23.32 ± 15.93: *U* = 4,689, *p* = 0.003), and higher levels of ptau181 (89.43 ± 68.40 vs. 65.21 ± 47.51; *U* = 2,947, *p* = 0.021) and *t*-tau (551.85 ± 363.56 vs. 418.53 ± 270.06; *U* = 2,873, *p* = 0.011). There were no statistically significant differences in age (71.50 ± 6.71 in MCI vs. 69.56 ± 7.63 in SCD; *U* = 3,159, *p* = 0.096), years of education (11.12 ± 4.73 vs. 11.07 ± 4.98; *U* = 3,674, *p* = 0.914), and sex (52.4 and 51.4% were women, respectively; X^2^ = 0.018, *p* = 1.0).

### 2.3 LASSI-L

Patients are instructed to memorize two sets of 15 words each, both characterized by semantic competition. These words are categorized into three common semantic groups: fruits, musical instruments, and articles of clothing. They are presented individually on cards at intervals of 4 s, and the patients read them out loud. Initially, the patients are presented with the first set of words, known as “list A.” After reciting all 15 words, they are given 60 s to freely recall them. Subsequently, semantic cues are provided for each category, allowing 20 s for each. To enhance encoding and storage, list A is then presented once more. Following this, another cued recall test is conducted (Cued Recall 2 list A [Cued A2]).

Upon completing the second cued recall for list A, a new set of 15 words (list B), also grouped into the same semantic categories, is introduced. The patient’s performance in free and cued recall is evaluated using a parallel procedure (Cued Recall 1 list B, [Cued B1]). List B is subsequently presented again, facilitating the examination of the failure to recover from proactive interference (frPSI) through another cued recall (Cued Recall 2 list B, [Cued B2]).

**Figure 1:**
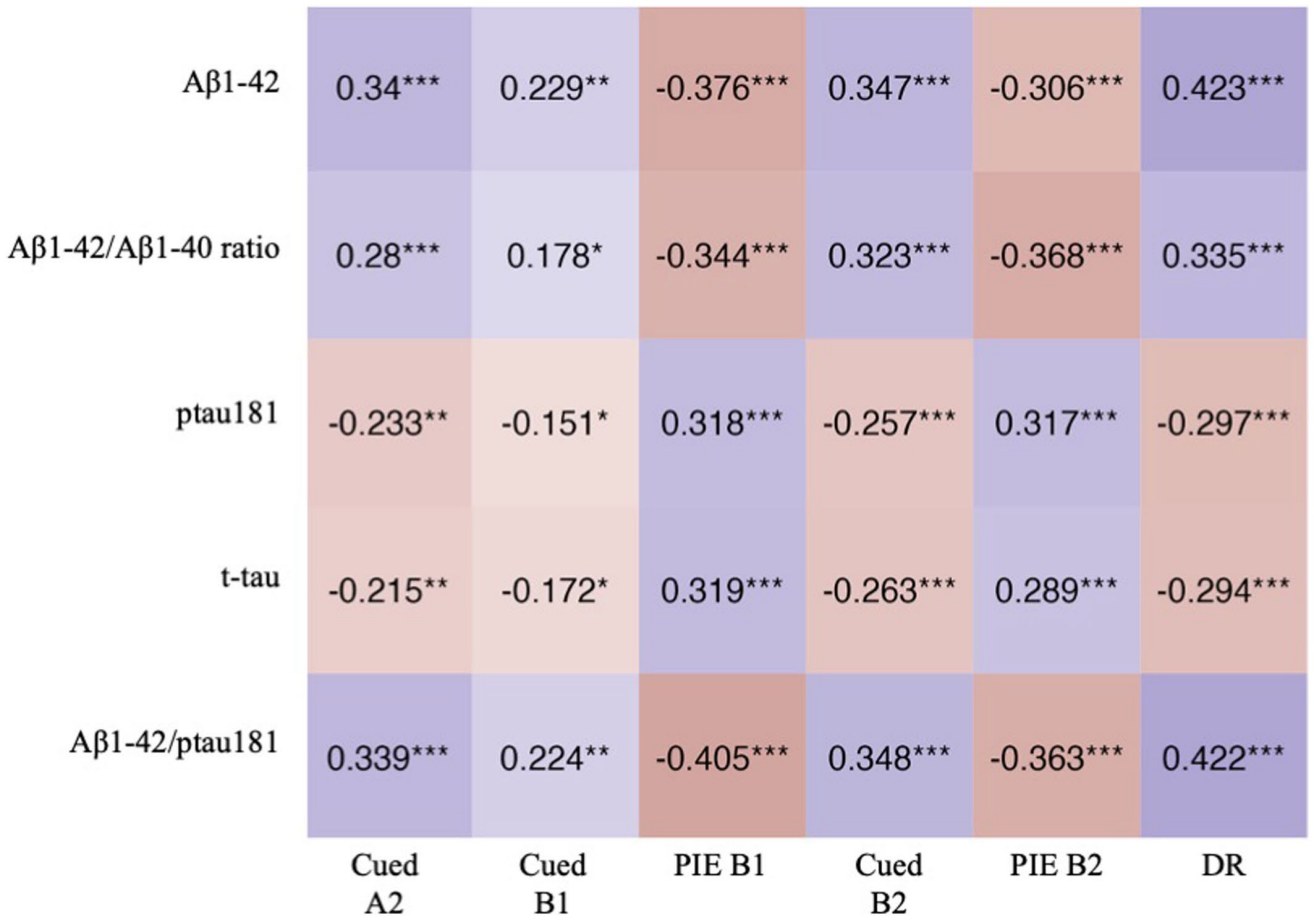
Heatmap showing correlations (Spearman’s correlation coefficient) between LASSI-L scores and CSF biomarkers. *p-value <0.05; **p-value <0.01; ***p-value <0.001.

After this, patients are asked to recall list A, both freely and with cues, in short-delay trials. Finally, after a 20-min delay, a delayed free recall test is conducted for both lists (Delayed Recall, [DR]). The scores are interpreted as follows: Cued A2 measures maximal storage, Cued B1 reflects the impact of proactive semantic interference, and Cued B2 represents recovery from proactive semantic interference (Loewenstein et al., 2016). The administration procedure and the scores of the LASSI-L are summarized in Figure 1.

We also registered the semantic intrusion errors made during each trial. Intrusions were categorized as intrusions from the other list (e.g., during the recall of list B, the patient evokes a word from the same semantic category but from the list A). Intrusions almost always comprise words from the competing list or semantic category. Curiel Cid et al. (2023b) has argued that the effects of intrusion errors may not be amply captured by those who have a low rate of responses in a particular recall trial. As such, we calculated the percentage of intrusion errors (PIE) as follows: [(total intrusion errors)/(total intrusion errors + total correct responses)] for Cued B1 (PIE-B1) and [(total intrusion errors)/(total intrusion errors + total correct responses)] CuedB2 (PIE-B2).

### 2.4 CSF analysis

The following biomarkers were determined in cerebrospinal fluid obtained by lumbar puncture: Aβ1-42, Aβ1-40, phosphorylated tau181 (p-tau181), and total tau (t-tau). These parameters were measured using a commercially available technique with CE-IVD marked (Lumipulse® Fujirebio). They were determined in the Lumipulse G600 II platform using the following reagents (product number 230336 and 230343 for Aβ1-42, product number 231524 and 231531 for Aβ1-40, product number 230350 and 230367 for ptau181, and 230312 and 230329 for total tau). Quarterly external quality controls from the University of Gothenburg (The Alzheimer’s Association QC program for CSF and blood biomarkers)^1^ were passed. We calculated the ratio: Aβ1-42/Aβ1-40, which has shown better diagnostic properties of amyloid deposition than Aβ1-42 (Hansson et al., 2019). The cut-off points were 0.068 for Aβ1-42/Aβ1-40 ratio, 723 pg./mL for Aβ1-42, 59 pg./mL for ptau181, 410 pg./mL for total tau. These cutoffs were provided by the manufacturer, and they were obtained from the participants of a study enrolling subjects with no cognitive issues and no progression to dementia after two years of follow-up (Wallin et al., 2016). According to the results, the manufacturer derived the values from percentiles 10th and 90th, and these were used as reference ranges. These cutoffs are similar to those obtained in independent cohorts comparing controls and patients with AD according to clinical criteria confirmed by follow-up and/or amyloid PET (Alcolea et al., 2019; Leitao et al., 2019). We also used 11.8 for Aβ1-42/ptau181, as calculated by Leitao et al. (2019) using the same platform.

### 2.5 Statistical analysis

Statistical analysis was conducted using IBM® SPSS version 26.0, JASP version 0.18.1 and Jamovi 2.3.21. The normality of the distributions was evaluated using the Kolmogorov–Smirnov test, revealing that all the LASSI-L scores, age, and years of education showed a non-normal distribution. Mann–Whitney’s *U* test was used to compare quantitative variables between two groups. Spearman’s correlation coefficient (rho) was used to measure the linear association between pairs of continuous variables. Correlations were interpreted as weak (<0.3), moderate (0.3–0.69), and strong (0.7–1). Effect sizes were evaluated using rank biserial correlation and regarded as small (0.10–0.29), medium (0.30 and 0.49), and large (≥0.50) (Lakens, 2013). Logistic regression analyses (backward stepwise conditional method) were conducted to investigate the relationship between LASSI-L scores and the likelihood of Aβ1-42/Aβ1-40 and ptau181 alterations. We introduced the raw LASSI-L scores and the age and years of education in the logistic regression analysis. Age and education were also entered due to their association with cognitive performance and amyloid deposition (Hönig et al., 2024). Bootstrapping with 1,000 resamples was used to obtain a more robust estimate of model coefficients and their associated estimated intervals. Hosmer-Lemeshow test was used to assess the performance of the model (*p* > 0.05 indicates good logistic regression model fit). Additionally, the model’s discrimination was assessed with accuracy and area under the curve (AUC).

## 3 Results

### 3.1 Correlation between LASSI-L scores and CSF biomarkers

Cued A2 showed moderate correlations with Aβ1-42 and Aβ1-42/ptau181, and weak correlations with ratio Aβ1-42/Aβ1-40, ptau181, and t-tau. The correlations with all the biomarkers were weak for Cued B1 but moderate for PIE-B1. Cued B2 showed moderate correlations with Aβ1-42, Aβ1-42/Aβ1-40 and Aβ1-42/ptau181, and weak with ptau181 and t-tau. PIE-B2 showed moderate correlations with Aβ1-42, Aβ1-42/Aβ1-40, ptau181, and Aβ1-42/ptau181, and weak correlation with t-tau. DR showed moderate correlations with Aβ1-42, Aβ1-42/Aβ1-40 and Aβ1-42/ptau181, and weak with ptau181 (Figure 1).

### 3.2 LASSI-L performance according to the amyloid status

In the whole sample, patients with amyloid deposition defined as a reduced Aβ1-42/Aβ1-40 ratio, showed lower scores in Cued A2, Cued B1, Cued B2, and DR, and higher scores in PIE-B1 and PIE-B2, evidencing worse performance. Effect sizes were medium for all the scores, except for Cued B1, that was small (Table 2).

**Table 2:** Comparison between groups according to their amyloid status (amyloid ratio) in the LASSI-L scores.

	Amyloid negative (*n* = 82)	Amyloid positive (*n* = 97)	Mann Whitney’s *U*	Rank biserial correlation
Age	67.12 ± 7.83	73.39 ± 5.00	2016**(<0.001)**	-
Years of education	11.92 ± 4.51	10.52 ± 5.01	4,624 (0.058)	-
Sex (% Females)	41 (50.0%)	52 (53.60%)	0.232 (0.630)*	-
Cued A2	10.48 ± 2.26	8.77 ± 2.49	2,480**(<0.001)**	0.37
Cued B1	5.32 ± 2.79	4.15 ± 2.18	3,048**(0.007)**	0.233
PIE-B1	39.98 ± 22.68	56.09 ± 20.43	1,632**(<0.001)**	0.39
Cued B2	8.90 ± 2.75	6.92 ± 2.61	5,546**(<0.001)**	0.39
PIE B2	20.17 ± 16.63	32.86 ± 19.82	1,685**(<0.001)**	0.37
DR	12.96 ± 6.51	8.03 ± 5.76	5,678**(<0.001)**	0.42

*X^2^-squared test. Cued A2, Cued Recall 2 list A; Cued B1, Cued Recall 1 list B; Cued B2, Cued Recall 2 list B; DR, Delayed Recall. PIE, Percentage of Intrusion Errors for Cued B1 (PIE-B1) and Cued B2 (PIE-B2). Statistically significant *p*-values are shown in bold.

### 3.3 LASSI-L performance in aMCI according to amyloid ratio and ptau181 levels

Patients in the aMCI group, 65 (63.10%) showed amyloid deposition according to the amyloid ratio. Patients with reduced amyloid ratio had worse performance on the LASSI Cued A2 (maximum learning capacity), Cued B2 (failure to recover from proactive semantic interference), DR and a higher percentage of intrusions in PIE-B1 and PIE-B2 compared with those with amyloid ratio within the normal limits. Effect sizes were medium for all these scores (Table 3).

**Table 3:** LASSI-L performance in MCI according to amyloid ratio, ptau181 levels, and Aβ1-42/ptau181.

	Amyloid ratio				ptau181				Aβ1-42/ptau181			
	Normal (*n* = 38)	Abnormal (*n* = 65)	Mann–Whitney’s *U* (*p*-value)	Rank biserial correlation	Normal (*n* = 48)	Abnormal (*n* = 55)	Mann–Whitney’s *U* (*p*-value)	Rank biserial correlation	Normal (*n* = 44)	Abnormal (*n* = 59)	Mann–Whitney’s *U* (*p*-value)	Rank biserial correlation
Cued A2	9.65 ± 2.12	8.10 ± 2.29	1,684**(0.002)**	0.36	9.04 ± 2.33	8.36 ± 2.32	1,501 (0.227)	0.13	9.45 ± 2.22	8.10 ± 2.28	1,689**(0.009)**	0.30
Cued B1	4.44 ± 2.17	3.65 ± 1.65	1,480 (0.090)	0.19	4.33 ± 2.01	3.56 ± 1.73	1,583 (0.078)	0.20	4.40 ± 2.10	3.55 ± 1.65	1,567 (0.069)	0.20
PIE-B1	45.01 ± 21.62	60.28 ± 17.69	555**(0.001)**	0.40	46.79 ± 20.43	61.63 ± 18.22	557**(<0.001)**	0.42	46.12 ± 21.05	60.65 ± 18.06	582**(0.002)**	0.39
Cued B2	7.81 ± 2.59	6.36 ± 2.36	1,608**(0.010)**	0.30	7.41 ± 2.50	6.45 ± 2.50	1,569 (0.097)	0.19	7.65 ± 2.53	6.33 ± 2.41	1,658**(0.015)**	0.28
PIE B2	24.92 ± 18.17	35.88 ± 18.37	618**(0.008)**	0.33	26.61 ± 18.45	36.43 ± 18.40	681**(0.017)**	0.30	25.60 ± 19.27	36.24 ± 17.52	653**(0.011)**	0.31
DR	10.73 ± 5.61	6.13 ± 4.48	1,688**(<0.001)**	0.46	9.77 ± 5.59	6.14 ± 4.62	1818**(<0.001)**	0.37	10.63 ± 5.46	5.74 ± 4.31	1952**(<0.001)**	0.50

Cued A2, Cued Recall 2 list A; Cued B1, Cued Recall 1 list B; Cued B2, Cued Recall 2 list B; DR, Delayed Recall. PIE, Percentage of Intrusion Errors for Cued B1 (PIE-B1) and Cued B2 (PIE-B2). Statistically significant *p*-values are shown in bold.

Considering ptau181 levels, 55 (53.3%) patients showed measurements above the cutoff. These patients showed lower scores in DR and higher intrusions on PIE-B1 and PIE-B2 compared to patients displaying ptau181 within normal limits. Effect sizes were medium for PIE-B1, PIE-B2, and DR (Table 3).

Using the Aβ1-42/ptau181 ratio, 59 (57.28%) showed abnormal levels. These patients showed lower scores in CuedA2, CuedB2, and DR, and higher intrusions on PIE-B1 and PIE-B2. Effects sizes were medium for CuedA2, PIE-B1, and PIE-B2, and large for DR (Table 3).

The percentage of agreement in MCI for the classification of patients according to amyloid ratio (altered vs. normal values) and ptau181 (altered vs. normal values) was 90.28%. The disagreements (10, 9.70%) were all patients considered altered according to amyloid ratio and within normal limits using ptau181.

The percentage of agreement between Aβ1-42/ptau181 ratio classification and amyloid ratio and between Aβ1-42/ptau181 ratio and ptau181 was 94.17% in both cases. The disagreements in the first case were 6 cases classified as abnormal according to amyloid ratio and normal with Aβ1-42/ptau181 ratio. In the comparison between Aβ1-42/ptau181 ratio and ptau181, the disagreements were 5 cases classified as abnormal according to Aβ1-42/ptau181 ratio and within normal limits using ptau181, and 1 case classified as abnormal according to ptau181 and normal using the Aβ1-42/ptau181 ratio.

### 3.4 LASSI-L performance in subjective cognitive decline according to amyloid ratio and ptau181 levels

In the group with SCD, 31 (43.05%) patients showed amyloid deposition according to the amyloid ratio. Patients with impaired amyloid ratio showed lower scores in Cued B2 and DR. Effect sizes were medium for Cued B2 and small for DR (Table 4).

**Table 4:** LASSI-L performance in SCD according to amyloid ratio, ptau181 levels, and Aβ1-42/ptau181.

	Amyloid ratio				Ptau181				Aβ1-42/ptau181			
	Normal (*n* = 41)	Abnormal (*n* = 31)	Mann–Whitney’s *U* (*p*-value)	Rank biserial correlation	Normal (*n* = 44)	Abnormal (*n* = 28)	Mann–Whitney’s *U* (*p*-value)	Rank biserial correlation	Normal (*n* = 46)	Abnormal (*n* = 26)	Mann–Whitney’s *U* (*p*-value)	Rank biserial correlation
Cued A2	11.31 ± 1.91	10.12 ± 2.41	804 (0.053)	0.244	11.34 ± 1.90	9.96 ± 2.42	807**(0.026)**	0.31	11.08 ± 1.97	10.30 ± 2.54	681 (0.328)	0.13
Cued B1	6.24 ± 3.10	5.32 ± 2.71	743 (0.219)	0.113	6.18 ± 3.03	5.32 ± 2.80	717 (0.243)	0.16	6.02 ± 3.04	5.53 ± 2.83	649 (0.552)	0.08
PIE-B1	35.15 ± 23.20	45.49 ± 23.35	295 (0.134)	0.171	34.70 ± 22.89	47.52 ± 23.23	258 (0.063)	0.30	35.29 ± 23.38	45.18 ± 23.59	272 (0.194)	0.22
Cued B2	9.90 ± 2.60	8.16 ± 2.73	857**(0.011)**	0.302	9.84 ± 2.63	8.07 ± 2.70	831**(0.013)**	0.34	9.60 ± 2.65	8.34 ± 2.87	739 (0.096)	0.23
PIE B2	15.97 ± 14.06	25.25 ± 21.72	297 (0.136)	0.166	15.47 ± 13.93	27.25 ± 21.81	253 (0.051)	0.31	16.40 ± 14.43	26.38 ± 22.60	261 (0.135)	0.25
DR	15.19 ± 6.57	12.25 ± 5.91	823**(0.033)**	0.267	15.25 ± 6.51	11.85 ± 5.79	816**(0.021)**	0.32	14.58 ± 6.65	12.76 ± 5.94	712 (0.18)	0.19

Cued A2, Cued Recall 2 list A; Cued B1, Cued Recall 1 list B; Cued B2, Cued Recall 2 list B; DR, Delayed Recall. PIE, Percentage of Intrusion Errors for Cued B1 (PIE-B1) and Cued B2 (PIE-B2). Statistically significant *p*-values are shown in bold.

Considering ptau181 levels, 28 (38.88%) patients showed measurements above the cutoff. These patients showed lower scores in Cued A2, Cued B2 and DR compared to patients displaying ptau181 within normal limits. There was a trend toward significance in PIE-B1 and PIE-B2. Effect sizes were medium for Cued A2, Cued B2, DR, PIE-B1, and PIE-B2. (Table 4).

Using the Aβ1-42/ptau181 ratio, 26 (36.11%) showed abnormal levels. There were no statistically significant differences in the LASSI-L scores between the groups displaying normal or abnormal Aβ1-42/ptau181 ratio.

The percentage of agreement in SCD between amyloid ratio and ptau181 was 95.82%. The disagreements (3, 4.16%) were all patients considered altered according to amyloid ratio and within normal limits using ptau181. The percentage of agreement between Aβ1-42/ptau181 ratio and amyloid ratio was 93.05%, and between Aβ1-42/ptau181 ratio and ptau181 was 94.44%. The disagreements in the first case were 5 patients classified as abnormal according to the amyloid ratio. In the comparison between Aβ1-42/ptau181 ratio and ptau181, the disagreements were 3 cases classified as abnormal according to the ptau181 and 1 case categorized as abnormal using the Aβ1-42/ptau181 ratio.

### 3.5 Logistic regression models for the prediction of CSF biomarkers abnormalities

Based on the aforementioned findings that DR was the score with the largest effect size, we estimated several logistic regression models based on this score, intrusions related to proactive semantic interference, and age (Table 5).

**Table 5:** Logistic regression analysis.

Variable	Coefficient (β)	Wald	Odds Ratio (OR)	95% confidence interval for OR	*p*-value
Aβ_1-42_/Aβ_1-40_ ratio below the cutoff					
DR	−0.103	12.674	0.904	0.853–0.955	<0.001
Age	0.133	20.512	1.142	1.078–1.210	<0.001
Constant	−8.162	14.789	0.008	-	<0.001
ptau181 above the cutoff					
DR	−0.100	8.683	0.905	0.847–0.967	0.003
Age	0.081	6.680	1.084	1.020–1.152	0.010
PIE-B2	0.021	3.828	1.021	1.00–1.042	0.050
Constant	−5.622	6.114	0.004	-	0.013
Aβ1-42/ptau181 ratio					
DR	−0.105	9.551	0.900	0.842–0.962	0.002
Age	0.084	7.171	1.088	1.023–1.157	0.007
PIE-B2	0.022	4.174	1.022	1.001–1.043	0.041
Constant	−5.751	6.312	0.003	-	0.012

Estimated coefficients and odds ratio. DR, Delayed Recall; PIE-B2, Percentage of Intrusion Errors for Cued Recall 2 list B.

**Figure 2:**
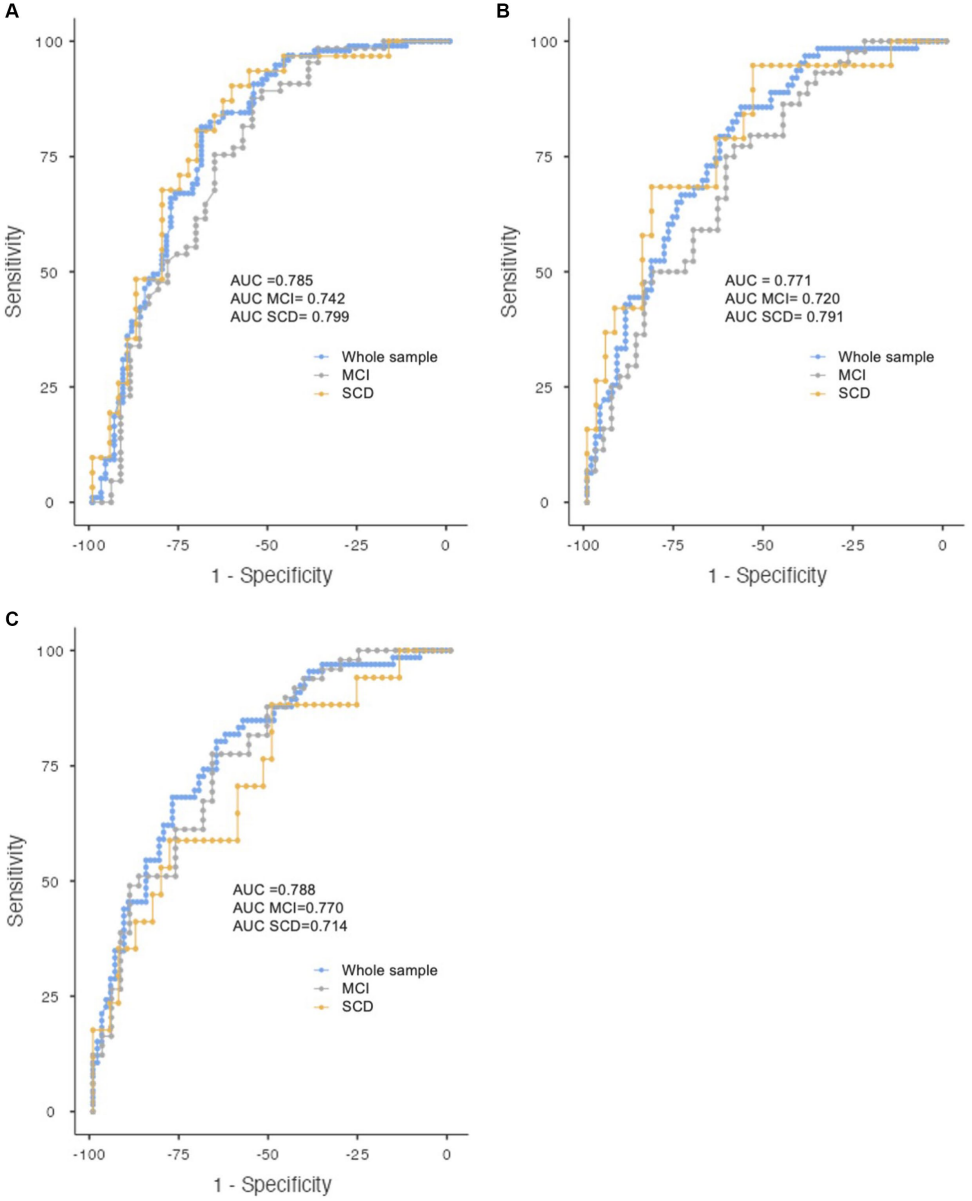
ROC curve showing the discrimination of the logistic regression models for abnormal amyloid ratio **(A)**, ptau181 **(B)**, and Aβ1-42/ptau181 **(C)** for the whole sample (blue), MCI (grey), and SCD (yellow).

The model for predicting amyloid deposition according to Aβ1-42/Aβ1-40 ratio (presence of amyloid vs. absence), included the variables DR and Age. The accuracy was 0.749 and AUC 0.785 (95% CI 0.716–0.855; *p* < 0.001). The Hosmer and Lemeshow Test was not significant (X^2^ = 10.264, *p* = 0.247), which means that the model fit the data well. For the MCI and SCD groups, the AUC was 0.742 (95% CI 0.635–0.848; *p* < 0.001) and 0.799 (95% CI 0.695–0.902; *p* < 0.001), respectively (Figure 2A).

The model for predicting increased ptau181 levels included the variables DR, age, and PIE-B2. The accuracy was 0.687 and AUC 0.771 (95% CI 0.696–0.845; *p* < 0.001). Hosmer and Lemeshow Test was not significant (X^2^ = 7.534, *p* = 0.480) (Figure 2B). For the MCI and SCD groups, the AUC was 0.720 (95% CI 0.614–0.825; *p* < 0.001) and 0.791 (95% CI 0.668–0.914, *p* < 0.001).

The model for predicting reduced Aβ1-42/ptau181 ratio included the variables DR, age, and PIE-B2. The accuracy was 0.714 and the AUC 0.788 (95% CI 0.715–0.860; *p* < 0.001) (Figure 2C). Hosmer and Lemeshow test was not significant (X^2^ = 9.225, *p* = 0.324). For the MCI and SCD, the AUC was 0.770 (95% CI 0.672–0.869; *p* < 0.001) and 0.714 (95% CI 0.568–0.861; *p* = 0.011).

## 4 Discussion

In this study, we aimed to determine the biological correlates of the LASSI-L. We included a large cohort of patients examined with CSF biomarkers as a proxy of brain amyloid and tau deposition using well-accepted CSF assays. These patients consulted for memory loss with no evidence of functional impairment. Patients were classified as aMCI or SCD, according to the results of the FCSRT and these individuals were biologically characterized with CSF biomarkers (Jack et al., 2016b).

The current findings yielded significant correlations between the LASSI-L scores and CSF biomarkers. According to previous studies using structural, functional, or molecular imaging, the most informative scores about the pathophysiology of AD have been Cued B2 and DR. In the present investigation, these same cognitive performance measures showed moderate correlations with Aβ1-42/Aβ1-40. Conversely, the magnitude of the correlation was lower for Cued A2, which represents maximum storage under a traditional approach of controlled learning and cued recall. These findings confirm that the assessment of memory with the LASSI-L and the use of the semantic interference paradigm is informative of the biological processes associated with the early stages of AD, especially amyloid deposition. The association with biomarkers was present for the raw measurement of amyloid and the Aβ1-42/Aβ1-40 ratio, which mitigate the effect of preanalytical confounders and CSF dynamics problems (Campbell et al., 2021).

The most discriminative score for the detection of amyloid ratio and ptau181 impairment was DR, followed by Cued B2, and Cued B2 PIE, as well Cued A2. These scores examine delayed recall at 20 min for the two lists, the recovery from proactive semantic interference, intrusions that occurred during the effects of proactive semantic interference, and maximum storage, respectively. This confirms that these processes are already impaired at early stages and associated with amyloid deposition. Among the different scores, DR outperformed the others because preservation of key memory processes is necessary to obtain a good performance (e.g., adequate encoding, learning under the effects of proactive interference, recall, etc.). However, it is worth mentioning that the behavior of the different scores showed some differences when analyzing the findings in aMCI and SCD. In the SCD group, only Cued B2 and DR showed statistically significant differences in the comparison of patients showing altered and preserved amyloid ratio and tau. Additionally, the effect size for Cued B2 was slightly better than DR, whereas the other scores were not statistically significant. This supports the hypothesis upon which the LASSI-L is founded, asserting that challenges in recovering from proactive semantic interference, as assessed by Cued B2, constitute a process impaired in the initial stages of AD.

Logistic regression models showed areas under the curve and accuracy levels acceptable for diagnosis. A combination of DR, intrusion errors on Cued B2, and age were the most common variables included in the models. It is worth noting that the variables selected and the capacity of prediction are very similar across amyloid ratio, ptau181 and Aβ1-42/ptau181 ratio, due to the interrelation between them. In this regard, the amyloid ratio is considered a surrogate of brain amyloidosis, while ptau181 is influenced by amyloid deposition, tau deposition and gray matter loss, depending on the disease stage (Lorenzini et al., 2022; Ossenkoppele et al., 2022). The ratio ptau181/Aβ1-42 (or the inverse quotient Aβ1-42/ptau181, as we preferred in this study to follow a previously validated cutoff with the same platform) has also been highly associated with amyloid PET status (Campbell et al., 2021). Importantly, ROC curves were similar in the aMCI and SCD. This suggests that the LASSI-L would also be sensitive in detecting amyloid deposition in patients with SCD according to the FCSRT. Furthermore, our results at the aMCI stage suggest that the LASSI-L seems to surpass the capacity of detection of amyloid positivity found in other studies with traditional tests in aMCI patients (Alves et al., 2021). However, specific studies directly comparing diagnostic performance are necessary. Overall, considering the area under the curve, future research combining more cognitive tests, genetic information, and/or other biomarkers (plasma, neuroimaging) to improve the accuracy of the statistical models is warranted.

These findings have important implications in clinical practice and research settings. LASSI-L could be used in clinical trials as an enrichment strategy and in clinical practice to guide the selection of patients before doing lumbar puncture or PET in order to increase the likelihood of AD. Additionally, a recent study has shown that the LASSI-L could also be combined with plasma biomarkers to improve the likelihood of amyloidosis in PET compared with plasma tau alone (Curiel Cid et al., 2023b). Overall, the recent developments of the most sensitive and non-invasive tools, including neuropsychological tests such as the LASSI-L and plasma biomarkers, suggest the convenience of reconsidering the screening strategies in the field. Despite the great development of imaging and fluid biomarkers, cognitive tools are necessary since the main objective of therapies is to delay or prevent cognitive decline. Thus, tools capturing early changes are needed. This should also be conducted considering the current diversity of many of the world’s populations, and in this regard, LASSI-L has also shown favorable cross-cultural properties (Curiel Cid et al., 2019).

Our study has some limitations. First, we used statistical models that assume linearity. However, the dynamics of the change of CSF biomarkers and the course in LASSI-L performance may require more complex models (Sepälä et al., 2011). Future studies using longitudinal designs and larger sample sizes are required to define the course of impairment in the LASSI-L and the relationship between LASSI-L performance and the different biomarkers and pathophysiological mechanisms (Krance et al., 2019). In this regard, the use of PET imaging may provide additional information by considering the topography of tau deposition and neurodegeneration. Similarly, the APOE status could also be useful in future studies. APOE4 status modulates the probability of amyloid deposition, although no direct effect on LASSI-L performance has been detected in a previous study (Zheng et al., 2022). Second, patients were attended in a memory clinic, although our center has direct access to primary care (Matias-Guiu et al., 2015b). However, our findings could not be applicable to other different settings (e.g., population-based studies). Third, neuropsychological examination (except LASSI-L) was one of the criteria for obtaining CSF biomarkers. For this reason, we did not evaluate the added value of any other neuropsychological test beyond the LASSI-L in the detection of amyloid or tau deposition.

In conclusion, our study supports the biological validity of the LASSI-L and its semantic interference paradigm in the context of the early stages of AD. The capacity of the test to detect early pathophysiological changes of AD suggests the usefulness of the test as a cognitive tool to guide the performance of invasive or expensive biomarkers (e.g., CSF, PET), or as an instrument to detect early memory loss and contextualize the results of more available but not specific biomarkers (e.g., plasma biomarkers).

## Data availability statement

The raw data supporting the conclusions of this article will be made available by the authors, without undue reservation.

## Ethics statement

The studies involving humans were approved by Comité de Ética de la Investigación con medicamentos (CEIm) Hospital Clínico San Carlos. The studies were conducted in accordance with the local legislation and institutional requirements. The participants provided their written informed consent to participate in this study.

## Author contributions

MV-S: Writing – review & editing, Writing – original draft, Visualization, Methodology, Investigation, Funding acquisition, Formal analysis, Data curation, Conceptualization. MG-M: Writing – review & editing, Investigation, Data curation. RC: Writing – review & editing, Methodology, Conceptualization. AD-Á: Writing – review & editing, Investigation, Data curation. IO-M: Writing – review & editing, Investigation, Data curation. CD-A: Writing – review & editing, Investigation, Data curation. MP-S: Writing – review & editing, Investigation, Data curation. JL-C: Writing – review & editing, Investigation, Data curation. MC: Writing – review & editing, Investigation, Data curation. JM-G: Writing – review & editing, Supervision, Investigation, Funding acquisition, Data curation. MD-C: Writing – review & editing, Supervision, Investigation, Funding acquisition. DL: Writing – review & editing, Methodology, Conceptualization. JAM-G: Writing – review & editing, Writing – original draft, Visualization, Supervision, Methodology, Investigation, Funding acquisition, Formal analysis, Data curation, Conceptualization.

## Footnotes

^1^ https://www.gu.se/en/neuroscience-physiology/the-alzheimers-association-qc-program-for-csf-and-blood-biomarkers
